# Lived Experiences of Family Members of Patients With Severe COVID-19 Who Died in Intensive Care Units in France

**DOI:** 10.1001/jamanetworkopen.2021.13355

**Published:** 2021-06-21

**Authors:** Nancy Kentish-Barnes, Zoé Cohen-Solal, Lucas Morin, Virginie Souppart, Frédéric Pochard, Elie Azoulay

**Affiliations:** 1 Famiréa Research Group, Medical Intensive Care Unit, Assistance Publique–Hȏpitaux de Paris, Saint Louis University Hospital, Paris, France; 2 Inserm Centre d'investigation clinique 1431, Centre hospitalier régional universitaire de Besançon, Besançon, France; 3 Assistance Publique–Hȏpitaux de Paris, Groupe Hospitalo-universitaire Nord, Hôpital Fernand Widal, Département de Psychiatrie et de Médecine Addictologique, Paris, France

## Abstract

**Importance:**

During the initial surge of the COVID-19 pandemic, family members were often separated from their loved ones admitted to intensive care units (ICUs), with a potential for negative experiences and psychological burden.

**Objective:**

To better understand the experiences of bereaved family members of patients who died in an ICU during the COVID-19 pandemic, from the time of hospital admission until after the patient’s death.

**Design, Setting, and Participants:**

This qualitative study used semistructured, in-depth interviews to collect experiences from bereaved family members of patients who died from severe COVID-19 in 12 ICUs during the first wave of the pandemic in France. Purposeful sampling was used to ensure the diversity of study participants with respect to sex, age, relationship with the patient, and geographic location. All data were collected between June and September 2020, and data analysis was performed from August to November 2020.

**Main Outcomes and Measures:**

Interviews were conducted 3 to 4 months after the patient’s death and were audio-recorded and analyzed using thematic analysis.

**Results:**

Among 19 family members interviewed (median [range] age, 46 [23-75] years; 14 [74%] women), 3 major themes emerged from qualitative analysis. The first was the difficulty in building a relationship with the ICU clinicians and dealing with the experience of solitude: family members experienced difficulties in establishing rapport and bonding with the ICU team as well as understanding the medical information. Distance communication was not sufficient, and participants felt it increased the feeling of solitude. The second involved the patient in the ICU and the risks of separation: because of restricted access to the ICU, family members experienced discontinuity and interruptions in the relationship with their loved one, which were associated with feelings of powerlessness, abandonment, and unreality. The third was regarding disruptions in end-of-life rituals: family members described “stolen moments” after the patient’s death, generating strong feelings of disbelief that may lead to complicated grief.

**Conclusions and Relevance:**

This qualitative study found that during the initial wave of the COVID-19 pandemic in France, bereaved family members described a disturbed experience, both during the ICU stay and after the patient’s death. Specific family-centered crisis guidelines are needed to improve experiences for patients, families, and clinicians experiences.

## Key Points

**Question:**

What is the experience of bereaved family members after the death of a loved one in an intensive care unit (ICU) during the first wave of the COVID-19 pandemic?

**Findings:**

In this qualitative study of 19 bereaved family members, participants reported difficulties in establishing a bond with the ICU team and maintaining a relationship with their loved ones during their stay in the ICU. They also described a feeling of “stolen moments” after the death of their loved one, generating strong feelings of disbelief that could potentially lead to complicated grieving.

**Meaning:**

These findings suggest that while adapting care practices and visiting policies is necessary given the public health menace, preventing families from seeing their loved ones altogether was highly detrimental: specific family-centered guidelines for crisis management are needed.

## Teaser

This qualitative study describes the experiences of family members of patients who died of COVID-19 in intensive care units (ICUs) in France.

## Introduction

During the initial surge of the COVID-19 pandemic, hospital visits were either banned or highly restricted, and family members were often separated from their loved ones admitted to intensive care units (ICUs).^1^ Relatives were no longer allowed to be at the patient’s bedside, which may have compromised their involvement in decision-making. Many clinicians felt that this situation was harmful both for patients and family members.^2,3^ In this context, communication with family members was considerably modified and relied primarily on distance communication—most often via telephone, sometimes using video conferencing—potentially leading to a decrease in the quality of communication, information, and sense of support.

After a death in the ICU, bereaved family members are at high risk of presenting symptoms that can negatively impact their quality of life, such as anxiety, depression, posttraumatic stress disorder symptoms,^4,5^ and complicated grief.^6^ Interestingly, witnessing terminal dyspnea and not being able to say goodbye to a loved one are factors associated with increased psychological burden among relatives.^6^ Thus, we speculated that the COVID-19 pandemic and the protective measures implemented in its wake may have exacerbated these negative experiences.^7^

Besides these potentially complex experiences in the ICU, previous research has reported that large-scale epidemics are associated with multiple simultaneous losses, related not only to death itself but also to the disruption in social norms, end-of-life rituals, and mourning practices.^8^ This may hinder the ability for individuals to connect with the deceased both before and after the death, thereby potentially increasing the risk of complicated grief.^9^

Considering the limited research in this field, learning from the current COVID-19 crisis is necessary. More specifically, it is important to achieve in-depth understanding of family members’ experience of the patient’s stay and end of life in the ICU during the pandemic to improve practices in the future and help develop specific family-centered guidelines in times of crisis. Qualitative research is useful to shed light on the subjective experience of bereaved family members, unearth their thoughts and feelings, and improve our understanding of their needs in the context of major health crises.^10,11^

 Our objective in this study was to better understand the experience of family members of patients with COVID-19 who died in ICUs, from the time of admission until after the patient’s death.

## Methods

This qualitative study was approved by the research ethics board of Sud Méditerranée. All study participants provided oral informed consent, including permission to publish quotes. Findings are reported in accordance with Standards for Reporting Qualitative Research (SRQR) reporting guideline.

### Design and Setting

This qualitative study is part of a larger quantitative multicenter study exploring the psychological burden experienced by family members of patients admitted to the ICU for COVID-19 during the initial surge of the pandemic in France (the BURDENCOV study). Briefly, participating family members were contacted 3 months after the patient’s discharge or death to take part in a telephone interview to complete a questionnaire describing their experience during the patient’s ICU stay, as well the Hospital Anxiety and Depression Scale and the Impact of Event Scale Revised (for symptoms of posttraumatic stress disorder). At the end of the call, family members whose relative died in the ICU were offered the opportunity to participate in the qualitative part of the study, a semistructured interview conducted a few weeks later over the telephone. Purposeful sampling was performed based on sex, age, relationship with the patient, and geographic location of the ICU so that interviewees were representative of the general population of relatives of patients in the ICU (ie, stratified sampling).

### Data Collection

Interviews were conducted between June and September 2020, ie, 3 to 4 months after the patient’s death. Owing to the large geographical study area and strict social distancing policies, telephone interviews were selected as the optimal method of data collection. Telephone interviews have been successfully used with bereaved relatives.^12^ We used a semistructured interview guide that included the following themes: ICU admission, ICU stay, life during the patient’s ICU stay, end-of-life care, funerals and rituals, and grief and mourning (eTable in the Supplement). Questions were open-ended, and participants were encouraged to explore issues they considered relevant. The interview guide was developed a priori by the investigators with the input of the psychologists in charge of the quantitative follow-up. Interviews were conducted in French, audio recorded, and professionally transcribed. Demographic data were self-reported and included sex, age, and relationship with the patient. Recruitment of new participants was interrupted when data saturation was reached, ie, when no new themes emerged from the interviews.^13^ Although saturation was achieved at the 17th interview, we conducted 2 additional interviews beyond saturation because these family members had already agreed to participate and had their interview scheduled.

### Data Analysis

Data analysis followed a 4-step process. First, 2 authors (N.K.-B. and Z.C.-S.) independently reviewed 8 randomly selected transcripts and identified key themes and concepts that occurred throughout the interviews using thematic analysis.^14^ Second, a preliminary codebook was developed through an iterative process that started with the systematic comparison of the investigators’ respective codes and ended when the 2 authors achieved consensus.^15^ Discrepancies were resolved by discussion with a third researcher (L.M.). In the third step, the 8 initial transcripts were recoded using this codebook, and after final consolidation, a single researcher (Z.C.-S.) proceeded with the coding of the remaining interviews. The codebook contained a total of 27 codes grouped into 3 underlying themes. Finally, themes and subthemes were defined and named, and relevant quotes were selected to document and illustrate each subtheme. Data analysis was performed from August to November 2020.

## Results

Among 12 ICUs located in 7 different regions of France, a total of 37 bereaved relatives were invited to participate. Among them, 9 declined, 9 could not be reached, and 19 agreed to participate in the qualitative study. The participants’ characteristics are presented in Table 1. Most participants were women (14 [74%] women), 8 participants (42%) were the deceased’s partner, and 8 participants (42%) were the deceased’s child. The median (range) age was 46 (23-75) years. Interviews lasted between 30 and 120 minutes. We derived 3 major themes from qualitative analysis: difficulty in building a relationship with the ICU clinicians and the experience of solitude; the risks of separation with the patient in the ICU; and disrupted end-of-life rituals and the feeling of “stolen moments” with the deceased. For each theme, we derived 3 or 4 subthemes (Box). A selection of representative quotes illustrating each theme is presented in Tables 2, 3, and 4.

**Table 1.** Characteristics of Study Participants and Deceased Family Members

Interview	Participant Characteristic				Deceased family member characteristic			ICU
	Sex	Age, decade, y	Relationship with the deceased	Family situation	Sex	Age, decade, y	ICU LOS, d	
1	Woman	20s	Daughter	Single, no children, was living with the deceased	Man	50s	16	1
2	Woman	60s	Wife	Now single, living alone	Man	70s	21	2
3		40s	Daughter	Married, 3 children	Man	70s	5	3
4	Woman	30s	Daughter	In a relationship, living alone, no children	Man	60s	6	4
5	Woman	60s	Wife	Now single, 2 children	Man	70s	6	5
6	Woman	20s	Niece	Single, no children	Man	60s	NR	1
7	Man	40s	Son	Married, no children	Woman	60s	13	6
8	Woman	70s	Sister	Living with mother, no children	Man	70s	18	4
9	Woman	40s	Wife	Now single	Man	50s	9	3
10	Man	70s	Husband	Now single, living with daughter	Woman	60s	7	7
11	Woman	30s	Daughter	Married, 3 children	Man	70s	14	7
12	Man	60s	Husband	Now single, living alone, 3 children	Woman	60s	19	8
13	Woman	20s	Daughter	Living with partner, no children	Woman	60s	6	7
14	Woman	70s	Wife	Now single, living alone, 2 children	Man	70s	15	9
15	Woman	40s	Daughter	Married, 1 child	Woman	70s	14	9 and 1
16	Woman	20s	Granddaughter	Living alone, no children	Woman	80s	67	10
17	Woman	60	Wife	Now single, living alone, 2 children	Man	60s	15	11
18	Man	30s	Son	Married, 2 children	Man	70s	5	12
19	Man	30s	Husband	Now single	Man	50s	15	12

Abbreviations: ICU, intensive care unit; LOS, length of stay; NR, not reported.

Box. **Themes and Subthemes Identified in Interviews**

**Difficulty in Building a Relationship With the ICU Clinicians and the Experience of Solitude**

**Building a Relationship Over the Telephone**

- Structured communication
- Communication vs information
- The importance of paraverbal communication

**Suspended in Time and Space by New Rules**

- Solitude
- A feeling of unreality
- An emotional roller coaster

**Meeting the ICU Team**

- Establishing trust
- Humanization
- Solving conflicts

**The Patient in the ICU: The Risks of Separation**

**Total Ban on Visits**

- Feeling powerless
- The ICU team as an intermediary

**Regular Visits Throughout the Patient’s Stay**

- Playing a role
- Continuity

**End-of-Life Visits Only**

- Abandonment and unreality
- The possibility of closure
- Frustration

**Disrupted End-of-Life Rituals and the Feeling of “Stolen Moments” With the Deceased**

**Before the Funeral**

- Dehumanization
- Not seeing the body: disbelief and ambiguity

**The Possibility or Impossibility of a Ceremony**

- For some, no ceremony
- New policies and lack of meaning

**Rituals and Grief**

- Classic rituals and adjustment to bereavement
- Inventing new rituals and moving on
- “Stolen moments” and barriers to grieving

Abbreviation: ICU, intensive care unit.

**Table 2.** Representative Quotes for Theme 1: Difficulty in Building a Distance Relationship With the ICU Clinicians and the Experience of Solitude

Quote No.	Subtheme	Interview No.
**Building a relationship over the telephone**		
1	“It was a contract that was made between the team and me: they had warned me from the start that they would call me every day at around 4 pm to check on my wife’s health.… It was a landmark for me.”	10
2	“They didn’t call back, we had to call back every time.… You can’t just leave people like that in ignorance, with their heart beating, their legs shaking, waiting for the phone call that will say that. Well, you can’t do that. It’s inhuman to do that.”	7
3	“We were in need of explanations, I can tell you. Because my husband went to the hospital on his 2 feet, although he wasn’t in good shape. Then it was just a nightmare, with no explanations.… So, then, they did take some time to help us understand, but it was too late. For me, it was too late. I didn’t trust them anymore.”	17
4	“The doctor called every day, but I didn’t get a feeling of how my husband was really doing. It was only technical information.”	14
5	“They really understood me, they showed empathy. They said, ‘We know it’s hard for you. This is a difficult situation. We’re doing the best we can with so many patients, but we know how you’ve waited for this call, so we want to take the time to explain and discuss things with you.’”	11
6	“The tone of the voice who says it, the way the subject is broached, that’s what’s important.”	12
**Suspended in time and space by new rules**		
7	“What was very brutal was that it was during the lockdown, and my brother and I were very, very alone. It was a very special time; we really were all alone.… Yes, we felt terribly isolated.”	15
8	“It was like being in a film, I didn’t understand what was going on. What’s this story? How can it be possible that he’s gone? How did he die? Really, even today, I just don’t understand.”	9
9	“It was an absolute nightmare, I was living to the rhythm of the ICU phone calls, with ups and downs because there was this Monday when things were going rather well, and then the next day when we were told it wasn’t going well.… It was awful, just awful.”	15
**Meeting the ICU team**		
10	“I felt comforted to see that he was really being looked after, he was fine and to actually see that reassured me. I think we were lucky that he was cared for by such competent people.”	11
11	“When I was there, I felt that my mother wasn’t just another patient, she was Mrs D. I felt that the nurse had a lot of empathy.”	13
12	“We went from black to white, because clearly, when on Friday we were told, ‘We’ll call the police if you come,’ and on Saturday, when I did go, the nurse said ‘If you wish I can come with you [to the patient’s room], and I can stay with you if you don’t want to be alone.’… She was just perfect. I couldn't have dreamed of a better nurse.”	13

Abbreviation: ICU, intensive care unit.

**Table 3.** Representative Quotes for Theme 2: The Patient in the ICU and the Risks of Separation

Quote No.	Subtheme	Interview No.
**Total ban on visits**		
1	“The greatest pain for me was the absence. It was such a cut, such a brutal break with him. It was unbearable. Unbearable.… I asked them if I could see him, but they said no. Not seeing each other? The fact that no one could be there to hold his hand and tell him we’re at his bedside, it was my brother’s sure death.”	8
2	“It’s a cocktail of sensations, there’s everything, sadness, anger, frustration, anxiety. You don’t know what to do. You’re bound hand and foot.”	7
3	“When we asked the nurse to stroke his arm or his forehead, to tell him we were there, I think she did do it because she would say, ‘Yes, we’ll do it.’ Even a doctor told us, the night when he died, ‘Don’t worry, I’m going to stay with him, I’m going to tell him that you’re here.’ That’s it, that’s all we had between us.”	8
**Regular visits throughout the patient’s stay**		
4	“We managed to go there 3 times. I couldn’t have imagined just dropping her off on Sunday and then never seeing her before she died. That would have been horrible. The separation would have been so much more violent; it was already very hard but if I hadn’t been able to see her again. I don’t know if I would have gotten over it.”	3
5	“I told her that we were waiting for her at home, that we were all praying for her, that we wanted her to come back.… I would just talk to her. I was able to touch her, I had gloves and all the right equipment, so I could put my hand on her and tell her that we loved her very much and that we still needed her.”	3
6	“We were there for him all the way, right up to when he died. We were able to talk to him, stroke him, and kiss him. For me, that was really, really important: to be able to look at his face and to see the parts that I knew so well and that hadn’t changed.”	14
**End-of-life visits only**		
7	“I wish my mother had died when she had her cancers, because then at least we could have been there with her until the very end, every day. I would have done anything to be with her. But that last week in the ICU, it’s as if I didn’t do my duty as a child, that was the hardest thing.… She must have felt so alone, so not cared for by her family.”	13
8	“I think it contributed to the nightmare, a feeling of complete unreality. For 1 month, I was with her all the time in my head. I knew she was in an ICU bed, and yet I couldn’t see her.”	15
9	“It was important to me: above all I needed to touch him. And then I needed to talk to him. It was really good to be able to come. It was life-saving for me.”	4
10	“It was so important to be there. At least he didn’t die alone.”	18
11	“I asked the doctor if I could come and see him. She said, ‘We don’t let families come except when we really have no hope anymore’… And what I said was, ‘But this is when he needs me at his side, now more than ever, not just when there’s no more hope. That’s just not enough.’”	4
12	“Not being able to see him, now that was a bad experience. The ban was really difficult, and even now, we find it difficult to accept. Months later, it still brings us pain.”	11
13	“I managed to say everything that I needed to say to him.”	2

Abbreviation: ICU, intensive care unit.

**Table 4.** Representative Quotes for Theme 3: End-of-Life Rituals and the Feeling of ‘Stolen Moments’ With the Deceased

Quote No.	Subtheme	Interview No.
**Before the funeral**		
1	“All the people who died from COVID[-19] were put naked in plastic body bags and then straight into the coffin, without even preparing the body! [Silence] And you can never see the person again. It’s over. I don’t know who made these decisions but it was difficult. It’s extremely violent.”	10
2	“Why were we not allowed to see the body? None of us could go to the morgue, to check that it was really him. We don’t even have a formal confirmation that it’s him. Not one of us has identified the body.”	6
**The possibility or impossibility of a ceremony**		
3	“We couldn’t attend the cremation. They just gave us the date and time. It was so hard, so very, very hard, very difficult.”	3
4	“We weren’t allowed to go to the crematorium. It’s a shame. Because of this, we feel that we couldn’t be with her until the very end.”	16
5	“It was appalling.… We weren’t allowed to do anything. We couldn’t touch the coffin, we couldn’t bring flowers, nothing at all. I feel like we abandoned him! He left the hospital all alone, in his coffin, with all the other bodies waiting. No real ceremony, nothing at all. It lasted 14 minutes, with just me and the kids, 9 people.”	2
6	“It was like, ‘Okay, the package is wrapped, hop, it’s over now!’… It’s not a moment I want to remember.”	1
7	“It wasn’t worthy of a funeral.… People don’t deserve to be buried like that.”	6
**Rituals and grief**		
8	“I fought for things to happen… And it felt good for everyone. And the battle I fought afterwards felt good for me, too. I managed to have a mass.… We scattered the ashes in the cemetery when I thought it wasn’t going to be possible.… My grandchildren were able to come, although they live in different regions. That was important, very important. We were able to pay tribute to him, to talk about him.”	15
9	“The funeral was broadcast using Zoom^a^ and about 100 people were able to follow the funeral.”	12
10	“I was given the day and the time of the cremation. That, in itself, was ultra important: it gave us a moment to share collectively. We said, ‘At 4 o’clock, we must all stop what we’re doing to think about him, it’s a way of being together.’ We organized a Skype^b^ [online meeting] so that we could all be strong together at the same moment.”	4
11	“I came home from the memorial service yesterday.… I can now say to myself, ‘Well, that’s it, that’s it, it’s over.’ It makes me cry, but at the same time, it’s a relief, too. I now feel like I can start something else.”	14
12	“With COVID[-19], it’s even harder, because you can’t just gather yourself close to your loved one’s coffin and say one last goodbye. It’s unreal, it’s unreal. It’s not possible.”	8
13	“I call it *the stolen moment*, and by that I mean the funeral rituals. For a civilized society, such as ours, these rituals are important. In a classic death, you can accompany the deceased.… But, here, we’re missing some fundamental steps in the system! My wife is alive, she goes to the hospital, she dies in the hospital, and then, on the following Friday, we’re scattering her ashes.”	10
14	“It’s hard to grieve. Sometimes, I tell myself she will come back, it’s not possible, we didn’t bury her. For me, she’s here, she’s somewhere. I have her things at home, I didn’t tidy anything up, I didn’t touch anything, and everything is here.”	3

^a^ Video conferencing software; Zoom Video Communications.

^b^ Video conferencing software; Microsoft.

### Theme 1: Difficulty in Building a Distance Relationship With the ICU Clinicians and the Experience of Solitude

#### Building a Relationship Over the Telephone

##### Structured Communication

Family members described their need of a supportive framework, for instance in the form of daily calls at set times by the same clinician when possible. When an ICU implemented a protocol for distance communication, family members’ expressed satisfaction (Table 2, quote 1). In other cases, family members experienced a lack of proactive regular communication with the ICU team (Table 2, quote 2). This deficit of communication was associated with a breakdown in the trust placed in the institution (Table 2, quote 3). None of the study participants communicated with the ICU via video calls.

##### Communication vs Information

For many family members, communication over the telephone was restricted to the ICU team giving information about the patient’s physical condition (Table 2, quote 4), leaving family members frustrated in terms of support and empathy. Only some family members experienced effective communication (Table 2, quote 5).

##### The Importance of Paraverbal Communication

The choice of words but also the tone, pitch, pacing and rhythm are fundamental in distance communication. Family members were sensitive to the quality of paraverbal communication (Table 2, quote 6): when adapted to the relatives’ emotions, nonverbal communication was experienced as soothing.

#### Suspended in Time and Space by New Rules

##### Solitude

Owing to lockdown and social distancing rules, family members were often alone while their relative was in the ICU. Loneliness was described as particularly burdensome (Table 2, quote 7).

##### A Feeling of Unreality

In looking back, family members reported having difficulty in believing that their experience was real. One respondent stated, “It was like being in a film, I didn’t understand what was going on. What’s this story? How can it be possible that he’s gone? How did he die? Really, even today, I just don’t understand” (Table 2, quote 8).

##### An Emotional Roller Coaster

As many family members could not visit the patient and fully understand what was going on, they could only rely on what clinicians said over the telephone. As the clinical trajectory of patients with COVID-19 was initially unknown, clinicians alternatively conveyed positive and negative messages (Table 2, quote 9), which could leave family members feeling disoriented.

#### Meeting the ICU Team

##### Establishing Trust

At a distance, family members tended to imagine the worst. Participants reported that being able to meet the ICU team and see the patient was soothing and generated trust (Table 2, quote 10).

##### Humanization

Participants felt that being able to witness the care provided transformed their perception of the ICU (Table 2, quote 11). For participants, seeing that the patient was cared for as a person and not just another COVID-19 case was vital.

##### Solving Conflicts

Communication over the telephone was perceived as less than optimal and sometimes generated tensions or even conflicts. Participants felt that being able to meet the clinicians face to face helped to ease the situation and comfort relatives (Table 2, quote 12).

### Theme 2: the Patient in the ICU and the Risks of Separation

Among the 12 ICUs in which the participants’ loved ones were hospitalized, there were 3 visiting policies: total ban on visits despite visits being exceptionally negotiated at the very end of life (3 ICUs), regular but limited visits throughout the patient’s stay (5 ICUs), and end-of-life visits only (4 ICUs). These policies were felt to strongly impact family members’ experiences.

#### Total Ban on Visits

##### Feeling Powerless

The total ban on visits was experienced not only as a break in the bond between the family and the patient, but also as a breaking point in the patient’s perceived chances of surviving (Table 3, quote 1). In all families, the ban was associated with a very strong feeling of powerlessness (Table 3, Quote 2).

##### The ICU Team as an Intermediary

In a situation of total ban on visitors, the ICU team had to play the role of intermediary between the family member and the patient, which was the only way to maintain a semblance of continuity (Table 3, quote 3). When family members felt that trust in the team was broken, continuity could become problematic.

#### Regular Visiting Throughout the Patient’s Stay

##### Playing a Role

Ability to visit the patient was perceived as a key element in the family members’ experience (Table 3, quote 4). Regular visits enabled family members to play their role in supporting and caring for the patient (Table 3, quote 5).

##### Continuity

Regular visiting also was associated with continuity at the end of life and a sense of closure (Table 3, quote 6). This possibility helped family members give meaning to the patient’s trajectory as well as to their own experience. Indeed, family members were able to witness and better understand the patient’s deteriorating condition that led to death.

#### End-of-Life Visits Only

##### Abandonment and Unreality

For family members of patients in ICUs that only permitted visits at the end of life, the initial ban was associated with a strong feeling of abandonment (Table 3, quote 7). For some participants, reflecting on the initial ban was experienced as unreal (Table 3, quote 8).

##### The Possibility of Closure

However difficult the initial restriction, the possibility of being with the patient at the time of death was paramount, since it helped both to accept the situation and to regain participants’ family role (Table 3, quote 9). Participants perceived not letting the patient die alone as essential (Table 3, quotes 10 and 13).

##### Frustration

When reflecting on their experience, all family members of a patient in an ICU that only permitted end-of-life visits declared that this unique visit was insufficient (Table 3, quotes 11 and 12). For example, one participant stated, “I asked the doctor if I could come and see him. She said, ‘We don’t let families come except when we really have no hope anymore’… And what I said was, ‘But this is when he needs me at his side, now more than ever, not just when there’s no more hope, that’s just not enough’” (Table 3, quote 11).

### Theme 3: Disrupted End-of-Life Rituals and the Feeling of “Stolen Moments” With the Deceased

#### Before the Funeral

##### Dehumanization

Health and safety guidelines not only kept families away from the dying person but also from the body of the deceased. Indeed, during the peak of the pandemic, relatives were forbidden access to the body: body bags were closed once and for all in the ICU. Many families described a dehumanizing experience associated with feelings of anger and injustice (Table 4, quote 1).

##### Not Seeing the Body: Disbelief and Ambiguity

As in disappearance cases, not seeing the body was felt to create doubt and uncertainty among bereaved family members (Table 4, quote 2). Not being able to officially identify the body was associated with profound ambiguity.

#### The Possibility or Impossibility of a Ceremony

##### For Some, No Ceremony

In some situations, ceremonies were impossible to organize (Table 4, quote 3). This absence of ceremony was associated with a feeling of guilt toward the deceased (Table 4, quote 4). All the familiar rituals, regardless of faith or religious practice, were thus missing.

##### New Policies and Lack of Meaning

New policies were set for ceremonies, such as an important restriction in the number of people present and the strict interdiction to touch the coffin—a rule criticized by many family members (Table 4, quote 5). These altered ceremonies lacked meaning, deprived family members of important symbolic moments, and deprived the deceased of a dignified tribute (Table 4, quotes 6 and 7).

#### Rituals and Grief

##### Classic Rituals and Adjustment to Bereavement

A very small number of families were able to organize a ceremony up to their expectations, and this was experienced as a great relief, since appropriate rituals can facilitate adjustment to bereavement. For example, one participant stated, “I fought for things to happen. And it felt good for everyone. And the battle I fought afterwards felt good for me, too. I managed to have a mass…. We scattered the ashes in the cemetery when I thought it wasn’t going to be possible …. My grandchildren were able to come, although they live in different regions. That was important, very important. We were able to pay tribute to him, to talk about him” (Table 4, quote 8).

##### Composing New Rituals and Moving on

In the face of numerous and drastic restrictions, some bereaved family members became proactive and found new ways of fulfilling shared rituals. Some filmed the ceremony live so that relatives could share the moment online (Table 4, quote 9). Some chose to all listen to the same music at the same time, whereas others preferred to share a moment of silence to commemorate their loved one (Table 4, quote 10). The possibility of rituals, whether classic or adapted, encouraged the bereaved to move on (Table 4, quote 11).

##### “Stolen Moments” and Grieving Disruptions

For other bereaved family members, incomplete ceremonies were experienced as disembodied and unreal (Table 4, quote 12). Many families expressed anger, as they felt deprived of an important ritual (Table 4, quote 13). These “stolen moments” were described as barriers to grieving (Table 4, quote 14).

## Discussion

In this qualitative study, we report how bereaved family members’ experiences of care, death, and mourning were disrupted during the first wave of the COVID-19 pandemic, both during the hospitalization in the ICU and after the patient’s death. Qualitative interviews make it possible to identify what was at the heart of the families’ experiences during the pandemic, namely difficulties in establishing rapport and bonding with the ICU team and in understanding the information, in coping with discontinuity and interruptions in the relationship with the patient, and in dealing with the feeling of “stolen moments” after the patient’s death (eFigure in the Supplement).

Over the last decades, research has shown that family members of patients receiving care in ICUs are at high risk of psychological burden.^16^ Having a loved one who died or was close to death and reporting poor communication with the ICU team are 2 major risk factors. Quality of communication with the ICU team has been found to be central in shaping family members’ experience, both during the patient’s stay and after death.^17,18^ Communication perceived as inconsistent, unsatisfactory, or uncomforting is associated with higher risk of post-ICU burden.^6,19^ The risk of posttraumatic stress disorder–related symptoms increases when relatives feel that the information given is incomplete.^4^

In the context of the COVID-19 pandemic, our study found that communication with family members was considerably altered and relied primarily on distance communication—namely over the telephone—lessening the quality of communication, information, and support. Families of patients in the ICU typically need to receive information repeatedly and in different ways, and they need to be able to ask questions continually and in real time. Even if telephone and video calls do permit contact with clinicians who can inform the family members of the patient’s situation, the failure to generate a bond between the family and the ICU team can create tension. Indeed, telephone and video calls are often restricted to technical information, rather than effective communication, which can leave the family feeling frustrated. High-quality communication includes verbal and nonverbal communication, active listening, and empathic statements.^20^ High-quality communication not only permits the family to understand the situation but also to feel accepted, supported, and trustful, which distant communication does not allow to the same extent.^21^ Our study found that a clear information strategy was helpful but insufficient. When possible, being able to see their loved one and the care environment and interacting with the members of the ICU team helped families to better understand the patient’s medical situation and feel confident that their loved one was comfortable and being well cared for.

Our study also reports that hospital visits were essential to family members in their relationship with their loved one and agrees with what family-centered research has taught us in the last 20 years: the presence of family members in ICUs should remain a priority.^22^ Family members have an important role to play,^23^ and their presence at the end of life is essential. Interestingly, the absence or rare presence of family members in the ICU during the pandemic has been reported as one the most difficult experiences for clinicians as well.^2^ As a consequence, family-centered guidelines in times of crisis are warranted, ensuring that the place of family members in the ICU and their role at the patient’s bedside are preserved.

For many family members, the notion of disappearance was felt strongly: the patient left home, was admitted to the hospital, and was never seen alive again, thereby adding even more trauma to an already traumatic experience.^24^ The lack of closure for these families was notable throughout the interviews. Indeed, the ability to comfort the patient at the end of life and to say goodbye allows family members to anticipate their separation, and these are steps of crucial importance in end-of-life care rituals.^25^ Then comes the time of grieving that is shaped by these practices. Separated families may not get closure if they cannot ask questions, cannot observe the ICU team’s effort to stabilize their loved one, and sometimes cannot be present during dying and death.^26,27^ Failure to address the needs of families may result in additional psychological burden. Enabling videoconference calls with dying patients may help relatives who cannot be present.^28^ However, research is necessary to confirm the benefits of this approach. Other ways of maintaining contact could also be considered, such as writing letters or using an ICU diary, depending on cultural beliefs, practices, and values.

Lastly, our study shows that the pandemic has altered the way people grieve. Families confirmed that funerals, burials, and services were pared down, postponed, or held remotely (and with very few persons present),^29^ and it was difficult to observe cultural or religious mourning practices. In fact, families were deprived of some of the most important rituals that normally occur following a death, suggesting that we are experiencing an anthropological breakdown relating to the way in which people experience dying, death, and grieving. In addition, owing to lockdown and social distancing, some family members felt isolated and alone to deal with their emotions and grief.^30,31^ This context may have an impact on families’ grieving, with a higher risk of developing prolonged grief. Not being able to say goodbye to their loved one, not being with the patient during dying and death, not seeing the deceased’s body, and barely being able to observe common rituals may generate a sense of disbelief and doubt that may hinder the grieving process.^6,32^ Not only can this create added burden, it can also cause anger, as expressed by family members who felt that they have been robbed of their loved one’s death. These results, combined with those of other quantitative and qualitative studies, such as a 2021 study by Hanna et al,^33^ are an opportunity to anticipate better practices if the pandemic continues or when we are confronted with new major health crises in the future. Readjustments to better take the bereaved family members’ fundamental needs into account will have to be made if we want to prevent serious harm due to complicated or even pathological grief later on.

### Limitations

Our study has several limitations. First, it was conducted solely in France, where care practices may not be representative of other countries, health care systems, and cultures. However, since similar measures (eg, restricted visiting policies, funeral restrictions, lockdown) were reported in most high-income countries, we believe that our results could help to understand relatives’ experiences in other countries. Likewise, we used a purposive sampling strategy to maximize the diversity of family members who participated in the study. However, participation in qualitative interviews was voluntary, thus creating a possible selection bias: family members with difficulties in (or reluctance to) expressing themselves or their experiences during the pandemic may have been omitted. Interviews were conducted early after the patient’s death, making it difficult to study the bereaved families’ experiences in the long term. Additionally, participants experienced losing a loved one during the first wave of the COVID-19 pandemic, at a time when hospitals were overwhelmed by the dramatic increase in cases. With time and practice, institutions have adapted to the situation, thus likely also changing bereaved family members’ experiences.

## Conclusions

Our qualitative study found that, in the midst of a major public health crisis, the erosion of family-centered care practices was associated with a dramatic impact on the experiences of family members of patients who died. While adapting care practices and visiting policies was undoubtedly necessary, given the nature of the threat, preventing families from seeing their loved ones altogether was also highly detrimental. To avoid traumatic experiences for patients, families, and clinicians, specific family-centered guidelines for crisis management are needed. Research in this field is required and could help develop adequate training for clinicians. Four avenues for improvement can be highlighted from our study. First, it is vital to safeguard the bond between families and patients by maintaining the possibility of family visiting. Second, high-quality communication between clinicians and families should be a priority, including video calls when possible, intervention of a facilitator responsible for supervising physician-family communication during the crisis, and other individualized approaches. Third, essential rituals at the end of life and immediately after death must be preserved in some form. Fourth, bereaved relatives should be provided with effective social support in times of lockdown and social isolation.

## Notes

Supplement. **eTable.** Interview Guide **eFigure.** Insight Into Bereaved ICU Family Members’ Experience During the COVID-19 Pandemic
