# Supplementary material for: Lived Experiences of Family Members of Patients With Severe COVID-19 Who Died in Intensive Care Units in France
Source: JAMA Netw Open. 2021 Jun 21;4(6):e2113355. doi: 10.1001/jamanetworkopen.2021.13355 (PMC8218069; doi:10.1001/jamanetworkopen.2021.13355)
Supplement: Supplement. — eTable. Interview Guide eFigure. Insight Into Bereaved ICU Family Members’ Experience During the COVID-19 Pandemic [file jamanetwopen-e2113355-s001.pdf]

Supplemental Online Content

Kentish-Barnes N, Cohen-Solal Z, Morin L, Souppart V, Pochard F, Azoulay E. Lived experiences of family members of patients with severe COVID-19 who died in intensive care units in France. *JAMA Netw Open*. 2021;4(6):e2113355. doi:10.1001/jamanetworkopen.2021.13355

eTable. Interview Guide
eFigure. Insight Into Bereaved ICU Family Members' Experience During the COVID-19 Pandemic

This supplemental material has been provided by the authors to give readers additional information about their work.

© 2021 Kentish-Barnes N et al. *JAMA Network Open.*

eTable. Interview Guide

**ICU admission**<br>Prompt: "First, could you tell me about your loved-one's ICU admission? And how did you feel at that time?"	• The onset of the illness and it's aggravation
	• Arriving at the hospital
	• ICU admission
	• Understanding of the situation
	• Emotions
**ICU stay and impact on personal experience**<br>Prompt: "Could you tell me about your experience of your relative's time in the ICU?"	• Length of stay and experience of temporality
	• The possibility of visiting the patient in the ICU and experience
	• The impossibility of visiting the patient in the ICU and experience
	• Relationship with the patient in this context
	• Communication with the patient in this context
	• Dealing with the risk of infection
	• Hopes and fears during the patient's ICU hospitalisation
	• Interactions and relationship with the medical staff
	• Interactions and relationship with the nursing staff
	• Difficult and testing aspects of this experience
**Daily life during the patient's ICU stay**<br>Prompt: " Could you tell me about your daily life at home at that time? How did you personally experience these moments at home?	• Family life, relationship with relatives and friends
	• Waiting, anxiety, uncertainty, loneliness
	• Media coverage of COVID and of the ICU environment
**End-of-life**<br>Prompt: "If you agree, I would like for you to talk about your loved-one's end-of-life?"	• Aggravation of the patient's clinical situation and temporality
	• Interactions with ICU team (attitudes, behaviour)
	• EOL information, discussions and preparation
	• The possibility of being present
	• The possibility of saying good-bye
	• Family support (partner, children, others)
	• Dying and death
	• End-of-life rituals
**The funeral**<br>Prompt: "Can you tell me about your relative's funeral and your experience during that time?"	• Organisation and difficulties due to the epidemic
	• Presence of family members and friends
	• The absence of those who could not come
**Grief and mourning**<br>Prompt: "Can you tell me about how your are doing today?"	• Social support and/or isolation/feeling of loneliness
	• Your experience today, when looking back
	• Taking care of yourself and looking towards the future

© 2021 Kentish-Barnes N et al. *JAMA Network Open.*

eFigure. Insight Into Bereaved ICU Family Members' Experience During the COVID-19 Pandemic

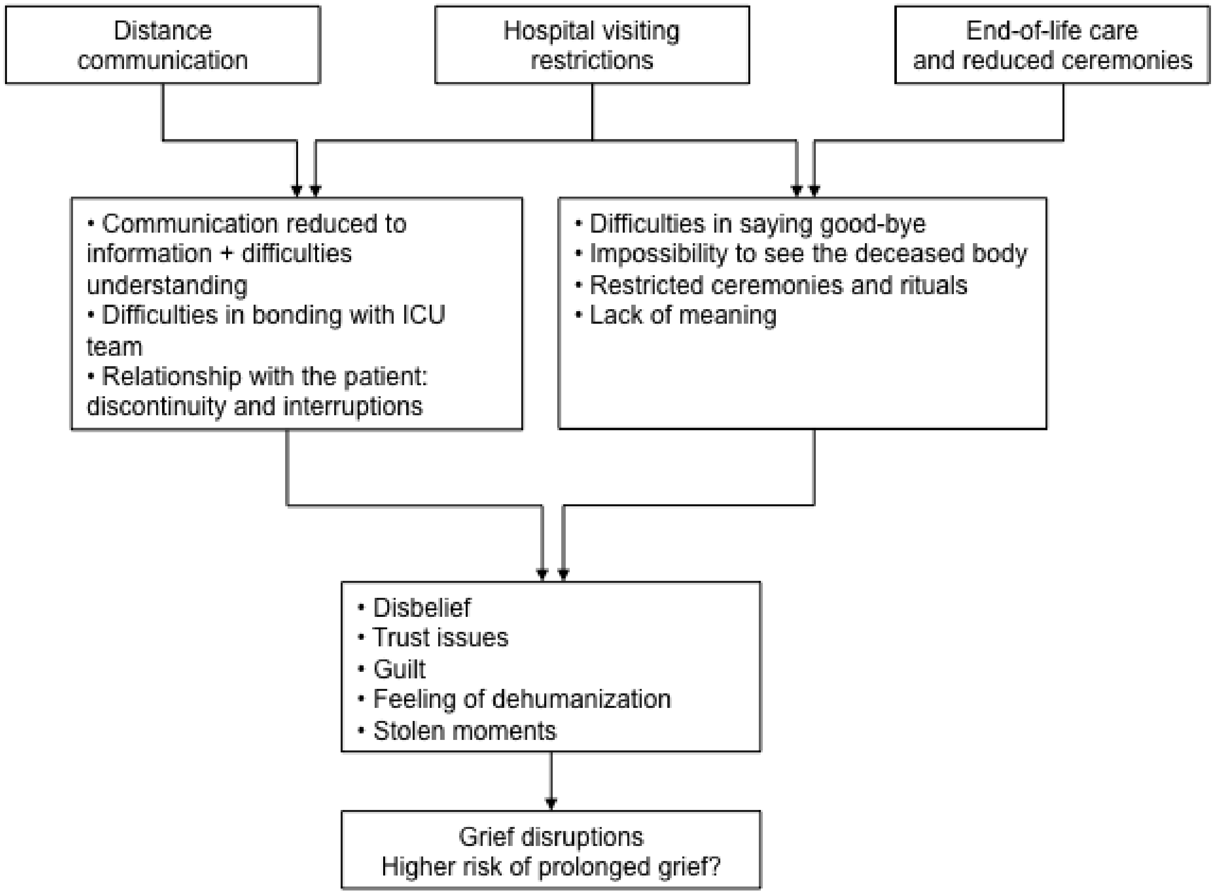

© 2021 Kentish-Barnes N et al. *JAMA Network Open.*
